# Supplementary material for: Emergent ferromagnetism with superconductivity in Fe(Te,Se) van der Waals Josephson junctions
Source: Nat Commun. 2023 Oct 23;14:6691. doi: 10.1038/s41467-023-42447-4 (PMC10593760; doi:10.1038/s41467-023-42447-4)
Supplement: Supplementary file 1 — Supplementary Information [file 41467_2023_42447_MOESM1_ESM.pdf]

*Supplementary Information for*

# Emergent Ferromagnetism with Superconductivity in Fe(Te,Se) van der Waals Josephson Junctions

Gang Qiu[1* †], Hung-Yu Yang[1*], Lunhui Hu[2], Huairuo Zhang[3,4], Chih-Yen Chen[5], Yanfeng Lyu[6], Christopher Eckberg[1,7,8,9], Peng Deng[10,1], Sergiy Krylyuk[3], Albert V. Davydov[3], Ruixing Zhang[2] and Kang L. Wang[1, †]

[1] *Department of Electrical and Computer Engineering, University of California, Los Angeles, CA 90095, USA*

[2] *Department of Physics & Astronomy, The University of Tennessee, Knoxville, Knoxville, TN 37996, USA*

[3] *Materials Science and Engineering Division, National Institute of Standards and Technology (NIST), Gaithersburg, MD 20899, USA*

[4] *Theiss Research, Inc., La Jolla, CA 92037, USA*

[5] *Department of Electrophysics, National Yang Ming Chiao Tung University (NYCU), Hsinchu, 30010, Taiwan*

[6] *School of Science, Nanjing University of Posts and Telecommunications, Nanjing, 210023, China*

[7] *Fibertek Inc., Herndon, Virginia 20171, USA*

[8] *DEVCOM Army Research Laboratory, Adelphi, Maryland 20783, USA*

[9] *DEVCOM Army Research Laboratory, Playa Vista, California 90094, USA*

[10] *Beijing Academy of Quantum Information Sciences, Beijing, 100193, China*

[†]Corresponding authors: Gang Qiu (gqiu@g.ucla.edu), and Kang L. Wang (wang@ee.ucla.edu)

[*]These authors contributed equally: Gang Qiu, Hung-Yu Yang

# Outline

**Supplementary Note 1: Additional information on air-free fabrication procedure and material characterization**

**Supplementary Note 2: Magnetic hysteresis data from another vJJ device**

**Supplementary Note 3: Critical current dependence on magnetic field history**

**Supplementary Note 4: Magnetic hysteresis loops at different temperatures**

**Supplementary Note 5: Understanding enhanced magnetism with interlayer spin-spin interactions**

**Supplementary Note 6: Field-dependent resistance of a superconducting reservoir (a bare FTS flake)**

**Supplementary Note 7: Additional discussion on the model Hamiltonian**

**Supplementary Note 8: Fraunhofer pattern measured with forward and reverse field scan directions**

**Supplementary Note 9: Fraunhofer pattern measured after the device experiences large magnetic field**

**Supplementary Note 10: Additional data on superconducting diode effect**

**Supplementary Note 1: Additional information on air-free fabrication procedure and material characterization**

The fabrication of FTS Josephson junctions (JJs) was done in an Ar-filled glove box via a standard pick-up and transfer technique [1]. The bottom and top FTS flakes were freshly exfoliated onto a $SiO_2$/Si substrate and a piece of polydimethylsiloxane (PDMS), respectively. The top FTS flake on the PDMS was then transferred onto the bottom FTS to form a vJJ with a pristine interface. A h-BN flake was exfoliated on PDMS and transferred on top of the FTS JJ to encapsulate the junction area. Finally, the h-BN/FTS/FTS heterostructure was picked up all at once using a layer of polypropylene carbonate (PPC) spin-coated on PDMS, and transferred onto a $SiO_2$/Si substrate with pre-patterned electrodes Au(95nm)/Cr(5nm) for electrical transport measurements.

We address that the interface of Fe(Te,Se) is extremely susceptible to the exposure to air, thus even following this precautious procedure to fabricate the junction device in an air-free environment, the device yield is still very low - only 5 out of 22 device we fabricated were superconducting. We also noticed that the superconducting device will even gradually degrade in the relatively inert environments after being stored in the glove box ($H_2O$ & $O_2$ $< 1$ ppm) for a week and becomes non-superconducting.

The Te/Se composition is determined by both Energy Dispersive X-ray Spectrometry (EDS) analysis and X-ray Diffraction (XRD) patterns. The EDS provides the more accurate estimation of Te/Se ratio. We performed the EDS scan during the cross-section TEM experiment. The resulting spectroscopy is shown in Fig. S1. The material composition of $Fe(Te_{0.58}Se_{0.42})$ can be derived from the spectroscopy analysis as shown in Table S1. From the literature, we confirm our material is in the topologically non-trivial regime with an inverted band at $\Gamma$ point [2,3].

Alternatively, we can also estimate the atomic ratio between Te and Se from the lattice constant measured from X-ray diffraction spectroscopy, as shown in Fig. S2. XRD experiments are carried out on the as-grown single crystal and a lattice constant of 6.04 Å along c-axis can be calculated from 001 peak position. We estimate a composition $Fe(Te_{0.57}Se_{0.43})$ by benchmarking with the reference data [4,5], which is very close to the EDS results.

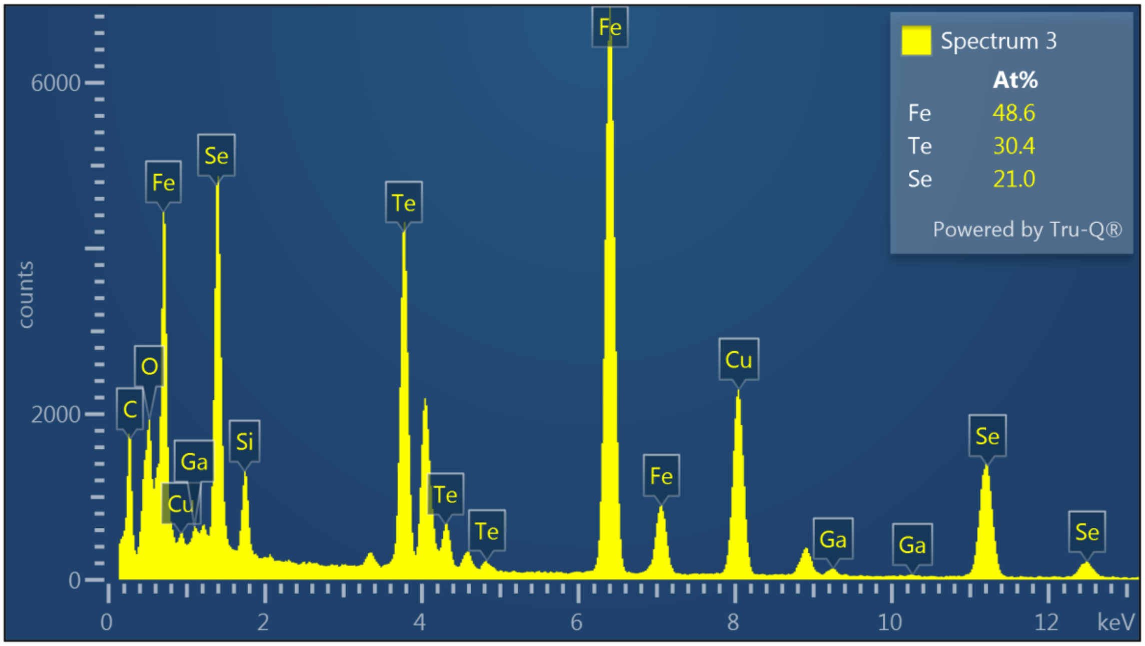


**Figure S1| Energy Dispersive X-ray Spectrometry (EDS) analysis of an Fe(Te,Se) flake.**

| **Spectrum 3** | | | | | | | |
|---|---|---|---|---|---|---|---|
| Element | Line Type | k Factor | k Factor type | Absorption Correction | Wt% | Wt% Sigma | Atomic % |
| Fe | K series | 1.166 | Theoretical | 1.00 | 32.86 | 0.22 | 48.56 |
| Se | L series | 1.703 | Theoretical | 1.00 | 20.12 | 0.25 | 21.03 |
| Te | L series | 2.001 | Theoretical | 1.00 | 47.02 | 0.28 | 30.41 |
| Total: | | | | | 100.00 | | 100.00 |

**Table S1| Energy Dispersive X-ray Spectrometry (EDS) analysis of an Fe(Te,Se) flake.**

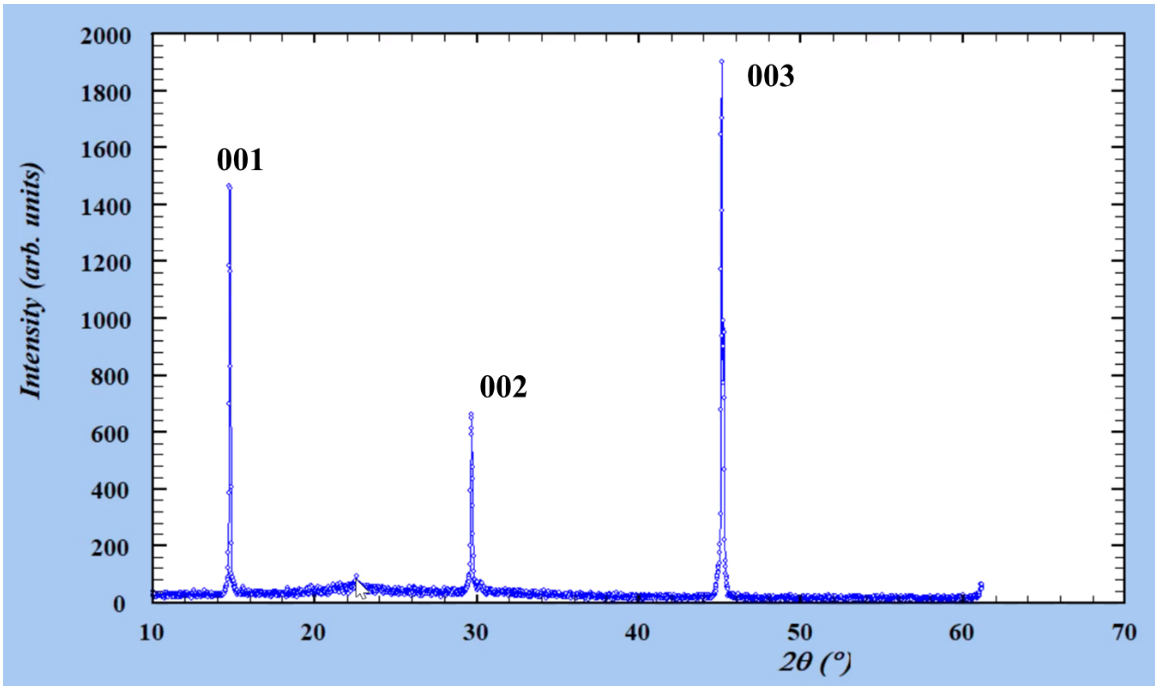


**Figure S2| X-ray Diffraction (XRD) spectroscopy of an as-grown Fe(Te,Se) single crystal.**

**Supplementary Note 2: Magnetic hysteresis data from another vJJ device (#F6)**

We present the transport data measured from another device (#F6). The general features of the junction behavior are reproducible as the device we presented in the main manuscript. The junction resistance also shows two-stage drops when cooling down, where the second stage can be quenched under a moderate field cooling (Fig. S3a). In this device, the junction interface may not be as pristine as the one we presented in the manuscript and thus there is a small but finite remnant resistance at the base temperature, as evidenced by a larger van der Waals gap from the cross-sectional TEM (Fig. S4). However, the main features were still preserved in this device. A similar hysteresis loop was also observed when sweeping magnetic field (Fig. S3b). We also notice that the hysteresis behavior is only observable with the presence of an in-plane magnetic field (i.e., perpendicular to the junction area), showing a strong magnetic anisotropy with an in-plane easy axis (Fig. S3c).

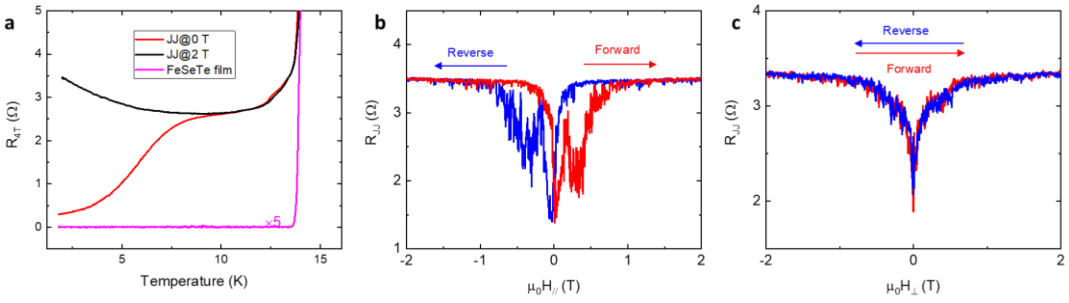


**Figure S3| Additional transport data from another vJJ device (#F6). a,** Temperature-dependent resistance of the junction under zero field (red) and 2T (black) field cooling, and the bare FTS flake (blue, 5x enlarged)**. b,** Magnetic hysteresis loops of the junction resistance under in-plane H (perpendicular to the junction area)**. c,** no magnetic hysteresis is observed under out-of-plane H (parallel to the junction area)**.**

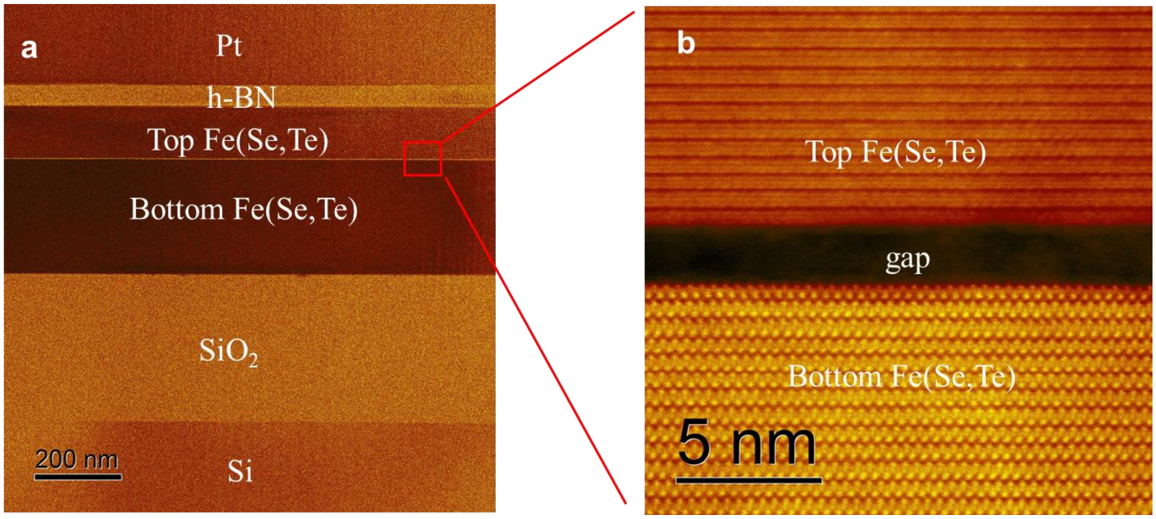


**Figure S4| Cross-sectional TEM data from another vJJ device.**

## Supplementary Note 3: Critical current dependence on magnetic field history

The critical current of the vJJ devices strongly depends on the magnetic field history, which can be explained by the surface electrons being magnetized and thus suppress the superconducting order. Fig. S5 demonstrates how the critical current is reduced from 330 µA to 100 µA after applying a magnetic pulse of 2 T. The magnetic field will align the spins at the interface and the magnetism will sustain after the field is moved. As a result, the superconducting states and consequently the critical current is strongly suppressed.

After performing a zero-field thermal cycle above Tc (in this case we heat the device to 25 K), the magnetic ordering is removed and the critical current is restored.

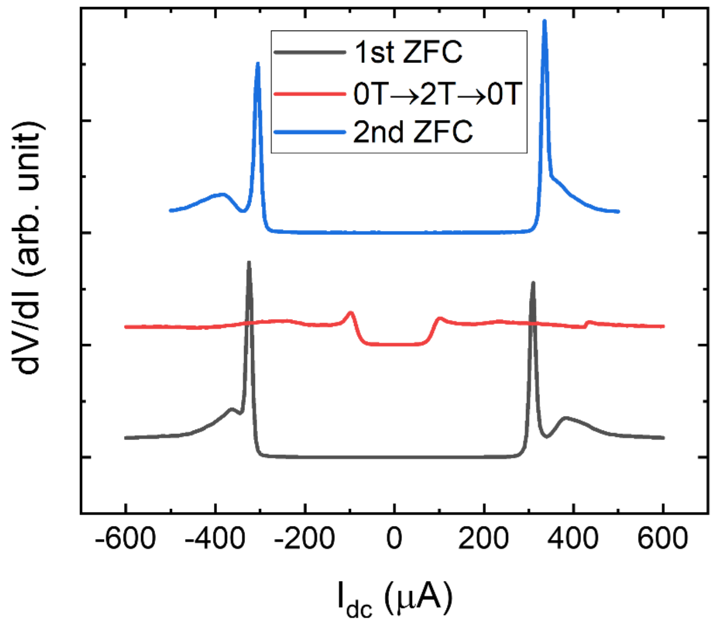


**Figure S5|** Critical current dependence on magnetic field history. Upon initial cooldown, the device shows a large 2 T magnetic field pulse, the critical current is reduced from 330 μA (black) to 100 μA. (red) The critical current is restored after a 2nd zero-field cooling procedure (blue).

## Supplementary Note 4: Magnetic hysteresis loops at different temperatures

The magnetic hysteresis loops were measured at different temperatures as shown in Fig. S6. The magnetic hysteresis loops smeared out at a temperature around 10 K, which is consistent with the superconducting critical temperature of the Josephson junction. This result points to strong correlations between ferromagnetism and superconductivity.

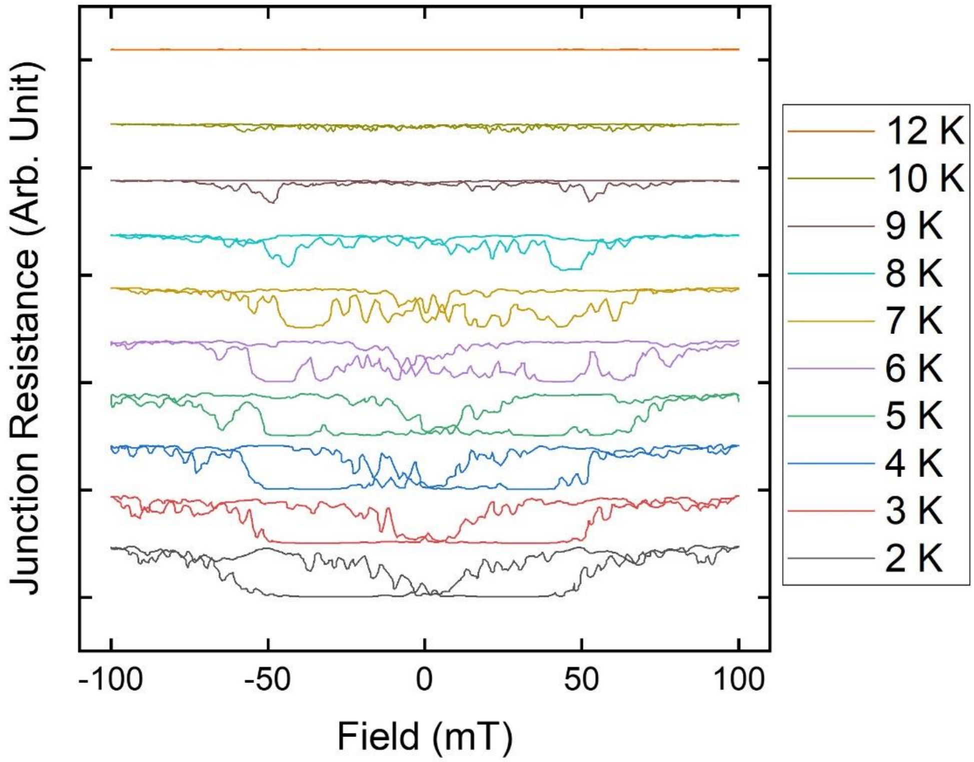


**Figure S6| Magnetic hysteresis loops of junction resistance measured at various temperatures from 2 K to 12 K.**

**Supplementary Note 5: Understanding enhanced magnetism with interlayer spin-spin interactions**

The spin-spin interaction between magnetization of top and bottom layers can be described by an effective Ginzburg-Landau theory,

$$F = \alpha_t(T)\left|\vec{M}_t\right|^2 + \beta_t\left|\vec{M}_t\right|^4 + \alpha_b(T)\left|\vec{M}_b\right|^2 + \beta_b\left|\vec{M}_b\right|^4 + \gamma\vec{M}_t \cdot \vec{M}_b$$

where we assume the second-order terms $\alpha_t(T) = \alpha_b(T) = \alpha_0\left(\frac{T}{T_M} - 1\right)$ with $\alpha_0 > 0$, and the fourth-order terms coefficients $\beta_t = \beta_b = \beta_0 > 0$. It also includes a bilinear coupling term, which is anti-ferromagnetic coupling ($\gamma > 0$) or ferromagnetic type ($\gamma <$

0), depending on the material details. The transition temperature is $T_M$ in the $\gamma = 0$ limit for each layer. Remarkably, this term could generally enhance the total magnetization (e.g, enhancing the transition temperature), like the Josephson effect. To understand this, we can take the symmetric solution $|\vec{M}_t| = |\vec{M}_b| = M_0$, and then the free energy becomes,

$$F = 2\alpha_0\left(\frac{T}{T_M} - 1\right)M_0^2 + 2\beta_0 M_0^4 + \gamma\cos\phi\, M_0^2$$

where the angle $\phi$ is the relative magnetization angle difference between top and bottom layers (as indicated in our experiments, magnetization is almost polarized in the $x - y$ plane). Therefore, the $\gamma$ term renormalizes the second-order term that determines the transition temperature as

$$2\alpha_0\left(\frac{T}{T_M} - 1\right) + \gamma\cos\phi \rightarrow (2\alpha_0 - \gamma\cos\phi)\left(\frac{T}{T_M'} - 1\right)$$

where the renormalized transition temperature $T_M' = T_M\left(1 - \frac{\gamma\cos\phi}{2\alpha_0}\right)$. Therefore, a negative $\gamma\cos\phi$ will enhance the transition temperature. In addition, the enhance magnetization at zero temperature can be obtained by

$$(2\alpha_0 - \gamma\cos\phi)M_0 + 2\beta_0 M_0^3 = 0$$

which gives rise to $M_0 = \sqrt{-\frac{2\alpha_0 - \gamma\cos\phi}{2\beta_0}}$. Notice that $\alpha_0 > 0$, $\beta_0 > 0$ and $\gamma\cos\phi < 0$, thus, this explains the enhancement of magnetization via the inter-layer spin-spin interactions, which is generally consistent with the symmetry argument mentioned in the main text. The reducing of symmetry leads to a lower ground-state free energy.

**Supplementary Note 6: Field-dependent resistance of a superconducting reservoir (a bare FTS flake)**

With the 8-terminal device geometry as shown in Fig. 1b, we were able to simultaneously monitor the resistance of two bare FTS flakes as Cooper pair reservoirs as we were

measuring the junction behavior (Fig. S7). The flakes do not show any deviation from superconducting states as expected since FTS have an extremely high critical field [6]. This is in sharp contrast to the junction behavior under a small magnetic field.

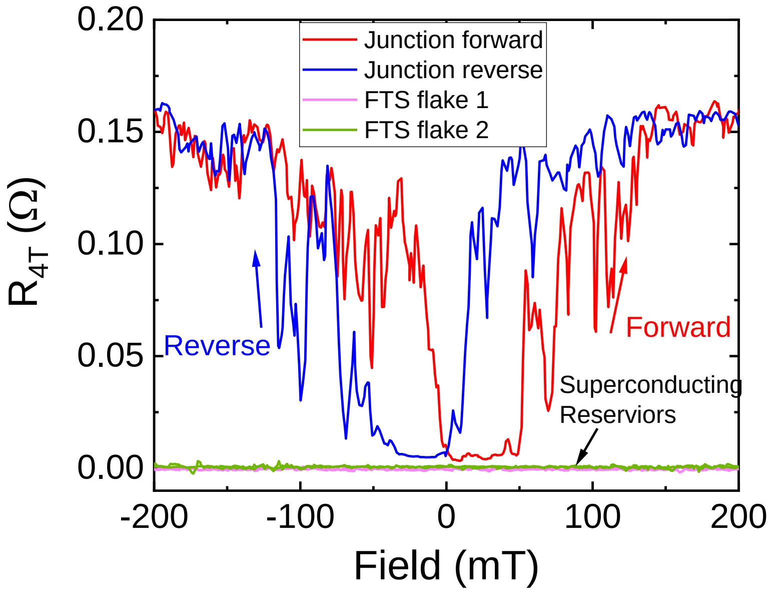


**Figure S7| Magnetic field vs vJJ and FTS flakes resistance.** The FTS flakes here refer to the two constituent superconducting reservoirs of the junction and the resistance values were recorded simultaneously.

**Supplementary Note 7: Additional discussion on the model Hamiltonian**

The above experimental observations directly restrict the low-energy effective model. Now we discuss the effective model Hamiltonian. Since all the above experimental facts consistently imply that these "unusual/puzzling" findings are attributed to the competition between surface magnetism and bulk superconductivity near the interface between top and bottom FTS flakes. Then, the model needs to fully characterize the interface electrons with both spin polarization/magnetization and proximity-induced superconductivity. FTS has been experimentally demonstrated to be topological with both Dirac surface states and metallic bulk bands around the Fermi energy [7]. Therefore, we ignore all the bulk bands that are trivially gapped out by an s-wave pairing potential below $T_c$, and study the hybridization of top and bottom surface Dirac states on the sample interface. The four-by-four Hamiltonian reads

$$H_{surf} = v_F(k_x\sigma_y - k_y\sigma_x)\tau_z + (t_0 + t_1k^2)\sigma_0\tau_x + (m_x\sigma_x + m_y\sigma_y + m_z\sigma_z)\tau_0 + \delta_\mu\sigma_0\tau_z - \mu\sigma_0\tau_0$$

where $v_F$ is the Fermi velocity of surface Dirac states, $t_0$ and $t_1$ are for the hybridization between top and bottom surface Dirac states, $\vec{m} = (m_x, m_y, m_z)$ is the spin magnetization, $\delta_\mu$ is the chemical potential shift of the top and bottom Dirac states, and $\mu$ is the overall chemical potential. Here $\sigma$ and $\tau$ are Pauli matrices for the spin and layer degrees of freedom. The basis is $\left(c_{t,\uparrow}(\vec{k}), c_{t,\downarrow}(\vec{k}), c_{b,\uparrow}(\vec{k}), c_{b,\downarrow}(\vec{k})\right)^T$, where the index $t, b$ labels the layers. The external in-plane magnetic field can increase the magnetization strength. Furthermore, the model clearly breaks both $P$ and $T$. Inversion symmetry is represented as $P = \sigma_0\tau_x$, which is clearly broken by nonzero $\delta_\mu$, and time-reversal symmetry $T = i\sigma_y\tau_0K$ with $K$ the complex conjugate is also broken by the spin magnetization $m_{x,y,z}$. Therefore, $H_{surf}$ is for the interface itinerant electrons and breaks both $P$ and $T$, as required by the experimental facts.

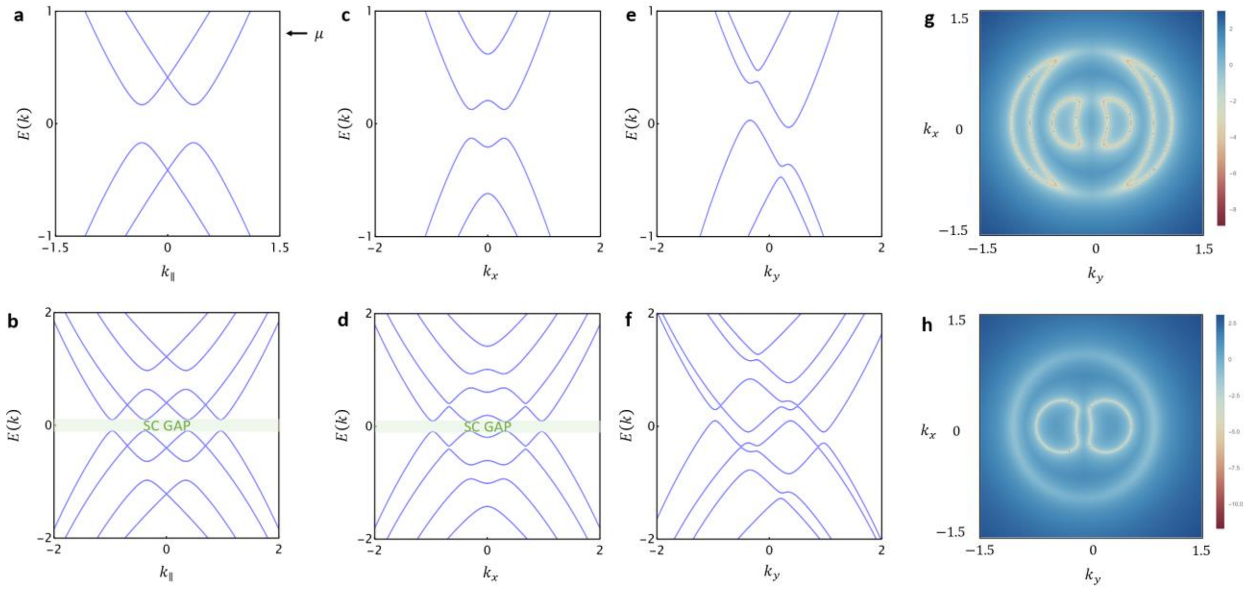


**Figure S8| The normal-state and superconducting band structures. a,** The normal-state bands with $m_x = m_y = m_z = 0$. **b,** The corresponding superconducting bands, where the green region represents the gap. **c,** and **e,** The normal-state bands with magnetization for $E(k_x, k_y = 0)$ and for $E(k_x = 0, k_y)$ **d,** and **f,** The corresponding superconducting bands along $E(k_x, k_y = 0)$ and for $E(k_x = 0, k_y)$. **g,** The partial Fermi surface based on BCS

pairing induced by gapless superconducting state due to the competition between magnetization and superconductivity. **h,** The partial Fermi surface based on FF pairing.

Next, we use this model to address the originality of the above discussed “unusual/puzzling” experimental findings. Before that, we first discuss the normal-state band structure by taking parameters as: $v_F = 1, \delta_\mu = 0.4, \mathrm{t}_0 = 0.1, t_1 = 0.5$ and $\mu = 0.8$ . The band structures in Fig. S8a are for the non-magnetic case ($T > T_c$) and (c-e) for the magnetic case ($T < T_c$), respectively. The surface Hamiltonian has a continues rotational symmetry about the z-axis, then we can choose $m_x = 0.2$ and $m_y = 0$ without loss of generality. Besides, we consider $m_x \gg m_z$ since the experimental data shows the magnetism is mainly concentrated in the x-y plane. Since $\delta_\mu$ breaks the inversion symmetry, it results in an effective 2D Rashba-like spin-splitting bands as shown in (a). And the Kramers degeneracy at $k_\parallel = 0$ is broken once the surface magnetism is turned on in (c) for $E(k_x, k_y = 0)$ and (e) for $E(k_x = 0, k_y)$.

The partial Fermi surface is a representative of gapless superconductors that have arc-like contours of zero-energy excitations with the same dimensionality as Fermi surfaces of normal states. It is a response to time-reversal symmetry breaking effects and can be induced by uniform current [8], magnetic field [9] or pseudo-magnetic field generated by time-reversal symmetry broken pairings [10]. It has been recently observed in experiments [11,12].

The surface magnetization in the bilayer FeTeSe system is a natural platform to explore the formation of partial Fermi surface. To show that, we turn on the proximity-induced s-wave superconductivity for the surface states. It has been shown that the fully gapped and isotropic gap function is induced in Ref. [7]. As a result, the Bogoliubov-de-Gennes (BdG) Hamiltonian with an isotropic spin-singlet pairing symmetry is given by

$$H_{\mathrm{BdG}} = \begin{bmatrix} H_{\mathrm{surf}}(k_x, k_y) & \Delta_0(i\sigma_y\tau_0) \\ -\Delta_0(i\sigma_y\tau_0) & -H^*_{\mathrm{surf}}(-k_x, -k_y) \end{bmatrix}$$

where the Cooper pairs have zero total momentum (BCS pairing), and we assume the pairing strength is the same for both layers. As we know, the Pauli limit can be violated when the finite-momentum pairing develops due to the interplay between magnetism and

superconductivity. For the normal states in the decoupling limit of top and bottom layers (i.e., $t_0 = t_1 = 0$ ), $\vec{m}$ breaks $T$ so that the energy $E(\vec{k}) \neq E(-\vec{k})$ for each layer. Practically, the in-plane magnetization tends to move Dirac cones away from the Γ point along the direction perpendicular to the in-plane magnetization direction, resulting in the movement of the Fermi surfaces in $k$-space. Therefore, the Cooper pairs from two electrons on the Fermi surface of each layer can carry a non-zero total momentum, which has lower free energy compared to that of the BCS pairing Hamiltonian. This explains the formation of finite-momentum pairing states when $|m| > \Delta_0$. Moreover, turning on the inter-layer coupling $t_0$ and $t_1$ does not affect the above argument, and the critical $\vec{m}$ depends on the material details. Given by the observation of diode effect in experiments, we will only focus on the Fulde-Ferrell (FF) pairing that is the so-called helical superconductivity in the literature, for which the basis can be chosen as

$$\Big(c_{t,\uparrow}(\vec{k}+\vec{q}), c_{t,\downarrow}(\vec{k}+\vec{q}), c_{b,\uparrow}(\vec{k}+\vec{q}), c_{b,\downarrow}(\vec{k} + \vec{q}), c_{t,\uparrow}^{\dagger}(-\vec{k}), c_{t,\downarrow}^{\dagger}(-\vec{k}), c_{b,\uparrow}^{\dagger}(-\vec{k}), c_{b,\downarrow}^{\dagger}(-\vec{k})\Big)^T$$

Therefore, the BdG Hamiltonian with the FF pairing becomes

$$H_{\mathrm{BdG}}(\vec{q}) = \begin{bmatrix} H_{\mathrm{surf}}(k_x+q_x, k_y+q_y) & \Delta_0(i\sigma_y\tau_0) \\ -\Delta_0(i\sigma_y\tau_0) & -H_{\mathrm{surf}}^{*}(-k_x,-k_y) \end{bmatrix}$$

In principle, the Cooper pairs' total momentum $\vec{q}$ needs to be solved self-consistently. However, one can simply take a good ansatz solution $\vec{q} \perp m_{\parallel}$ and $|\vec{q}| = \frac{1}{v_F}|m_{\parallel}|$ for a relatively large in-plane magnetization $\vec{m}_{\parallel} = (m_x, m_y)$, based on the previous self-consistent results [13,14]. Note that the Larkin-Ovchinnikov (LO) pairing breaking the lattice translational symmetry could also exist in the superconducting phase diagram, which will not be considered here since there is no direct experimental evidence for the LO phase.

With the model, then we discuss the quasi-particle spectrum of $H_{BdG}(\vec{q})$ by using $\Delta_0 = 0.1 < m_x$. We first focus on the case where the BCS pairing dominates, namely, by setting $\vec{q} = 0$. It reaches the Pauli limit when $|\vec{m}| \approx \Delta_0$ for a BCS superconductor. And the corresponding quasi-particle spectrum is shown in Fig. S8d for $E(k_x, k_y = 0)$ and S8f for $E(k_x = 0, k_y)$. We find that it becomes gapless along the $k_y$ axis, forming the partial

Fermi surface even through it is still in the superconducting state (see the fully gapped spectrum along the $k_x$ axis). The entire partial Fermi surface in the $k_x - k_y$ planes is shown in Fig. S8g, where two pairs of partial Fermi surfaces appear as required by the mirror symmetry. More generally, we can also analytically derive the gapless criteria for the $m_z = 0$ case is just $|m_x| \geq \Delta_0$. This can be shown from the spectrum of $H_{\mathrm{BdG}}$,

$$E_{k_x=0,k_y} = \pm m_x \pm \sqrt{\Delta_0^2 + \left(\sqrt{t_k^2 + \left(\mp v_F k_y + \delta_\mu\right)^2} \pm |\mu|\right)^2}$$

which have zeros once $|b_x| \geq \Delta_0$ and the corresponding $k_y$ can be solved from $\sqrt{t_k^2 + \left(\mp v_F k_y + \delta_\mu\right)^2} = |\mu|$ with $t_k = t_0 + t_1 k_y^2$. Please notice that a conventional BCS superconductor, $m_x = \Delta_0$ is approaching the Pauli limit, which makes it difficult in observing the partial Fermi surface in real materials. However, this cannot be applied to our case, because $\Delta_0$ is induced from the bulk superconductivity via an internal proximity effect. The pairing gap of bulk states is about three times larger than $\Delta_0$, ensuring the superconducting phase for the surface states. This means the bulk states are a fully gapped superconductor, while the surface states have partial Fermi surfaces.

As for the FF phase, we show that the partial Fermi surface still exists in Fig. S8h for the same parameter $\Delta_0 = 0.1 < m_x = 0.2$. Differently, only one pair of partial Fermi surface shows up because the other one got fully gapped by the FF pairing. Therefore, the formation of partial Fermi surface can be achieved on the interface of the bilayer FTS system, which in turn explains the tiny but finite resistance observed in experiments. Note that a clean gapless superconductor with partial Fermi surfaces is still a perfect conductor with zero resistance at zero temperature. However, the resistance due to the scattering of states on the partial Fermi surfaces may take place at low temperatures.

**Supplementary Note 8: Fraunhofer pattern measured with forward and reverse field scan directions**

The Fraunhofer patterns were measured after zero-field cooling and only a small magnetic field region was scanned (smaller than the coercive field of the surface ferromagnetism).

This ensures no long-range ferromagnetic ordering is established in the device. As a result, the forward and reverse scans produce almost identical patterns with no observable hysteresis.

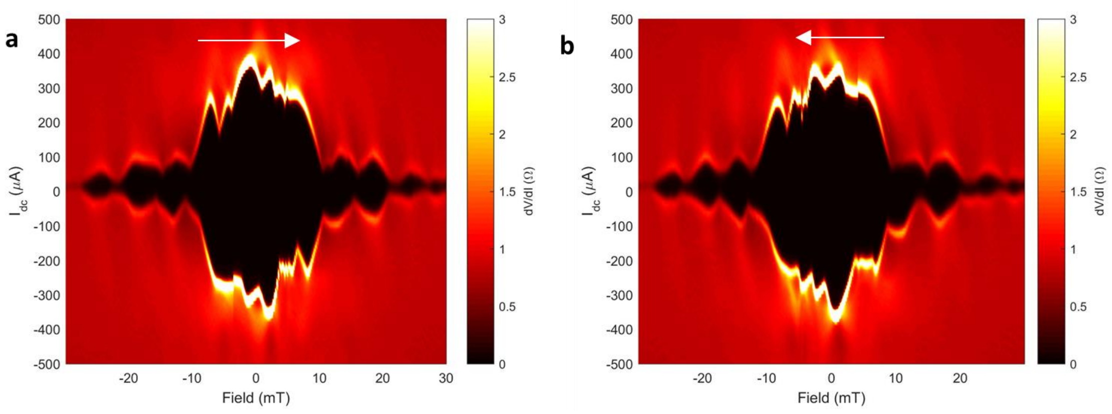


**Figure S9|** Fraunhofer pattern measured with forward and reverse field scan direction in a small magnetic field window. **a**, forward scan direction. **b**, reverse scan direction.

**Supplementary Note 9: Fraunhofer pattern measured after the device experiences large magnetic field**

On the contrary, when recording the Fraunhofer pattern in the same device in a larger magnetic window, the surface magnetism will cause hysteresis in the superconducting states. the superconducting interference patterns are also ill-developed as they are overwhelmed by noises likely caused by flux jumps and domain motions.

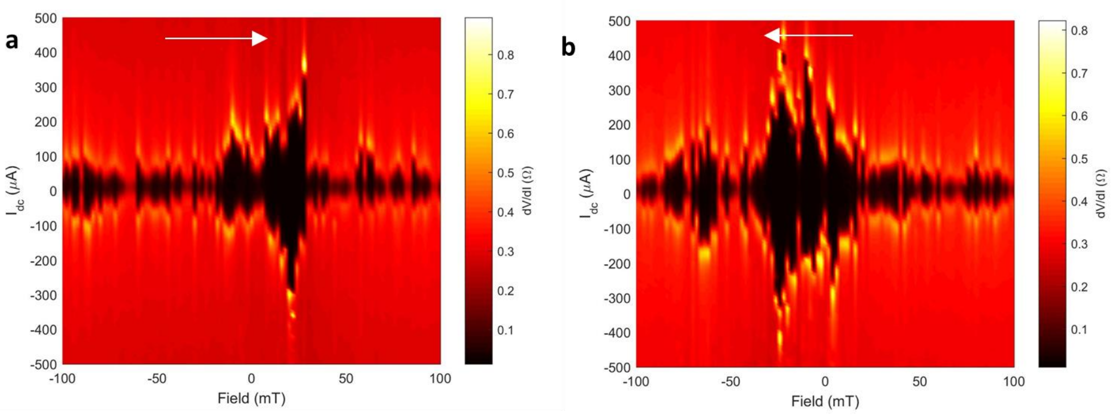

**Figure S10|** Fraunhofer pattern measured with forward and reverse field scan direction in a large magnetic field window. **a**, forward scan direction. **b**, reverse scan direction.

## Supplementary Note 10: Additional analysis on the superconducting diode effect

We provide further analysis on the stochasticity of field-free diode effect. We show the statistic distribution of critical current measurements in Fig. S11a. The critical current is defined as the DC current at which the differential resistance reaches a peak. Both positive and negative critical current were taken at the outbound sweep direction to avoid the Joule heating effect and compensate the delay from line filtering. We can see a much broader distribution of critical current between thermal cycles where the magnetization is randomly reset with thermal cycles. We also plot the critical current measurements with thermal cycles as a function of repletion numbers (Fig. S11b), and there is no obvious drift and diminishing of SDE with time, which means this stochastic field-free SDE is a robust and reversible process. It should also be pointed out that the average values of positive and negative critical current are not equal. This may be caused by a remnant field due to trapped flux from the superconducting coil. It has been shown that a few Oersted of magnetic field is sufficient to induce the SDE. Therefore, it is reasonable to conjecture that this remnant field will cause a systematic shift in the average value. However, the SC magnet was put in the "persistent" mode throughout this entire measurement, hence the remnant field will remain unchanged and should not produce such random SDE. Therefore, we can conclude the SDE is an intrinsic effect from this material.

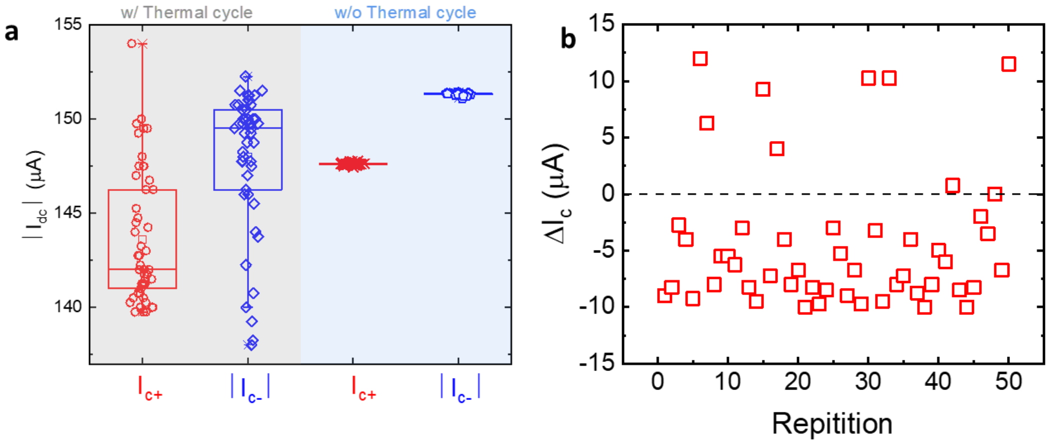

**Figure S11| Statistics of critical current and field-free diode effect. a,** Distribution critical current with (left panel) and without (right panel) thermal cycles between measurements. The boxs represent 25% and 75% range and the lines mark the median value. **b,** The difference between positive and negative critical current ($\Delta I_c = I_{c+} - I_{c-}$) plotted in the measurement sequence.

Finally, as a proof-of-concept, we demonstrate the current rectifying function of our SDE (see Fig. S12). We apply a small magnetic field of 10 mT to deterministically magnetize the vJJ and source a current of ± 100 µA. The device resistance switches between a high resistance state and low resistance states. By reversing the magnetic field direction, the polarity of the SDE is also changed. The on-state of the diode does not fully drop to zero resistance, due to the soft SC gap of FTS, where quasiparticles contribute to a finite resistance.

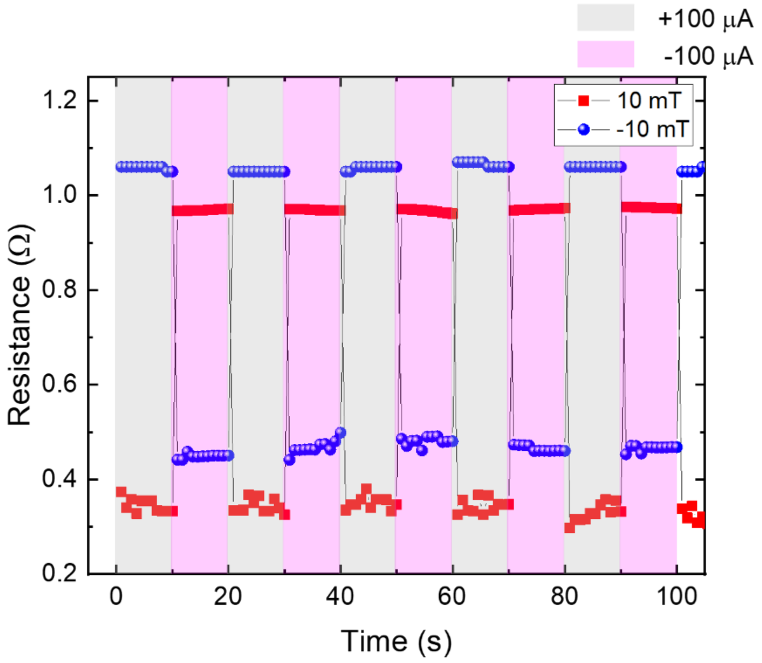


**Figure S12| Current rectifying function of superconducting diode effect.** A low magnetic field of ± 10 mT is applied to assist in the deterministic diode effect. The device operates at a DC current of ± 100 µA.

## Supplementary References


1. Wang, L. *et al.* One-Dimensional Electrical Contact to a Two-Dimensional Material. *Science* **342**, 614-617, doi:doi:10.1126/science.1244358 (2013).
2. Li, Y. *et al.* Electronic properties of the bulk and surface states of $Fe_{1+y}Te_{1-x}Se_x$. *Nature Materials* **20**, 1221-1227, doi:10.1038/s41563-021-00984-7 (2021).
3. Shi, X. *et al.* $FeTe_{1-x}Se_x$ monolayer films: towards the realization of high-temperature connate topological superconductivity. *Science Bulletin* **62**, 503-507, doi:https://doi.org/10.1016/j.scib.2017.03.010 (2017).
4. Fang, M. H. *et al.* Superconductivity close to magnetic instability in $Fe(Se_{1-x}Te_x)_{0.82}$. *Physical Review B* **78**, 224503, doi:10.1103/PhysRevB.78.224503 (2008).
5. Imai, Y., Sawada, Y., Nabeshima, F. & Maeda, A. Suppression of phase separation and giant enhancement of superconducting transition temperature in $FeSe_{1-x}Te_x$ thin films. *Proceedings of the National Academy of Sciences* **112**, 1937-1940, doi:doi:10.1073/pnas.1418994112 (2015).
6. Tsurkan, V. *et al.* Physical properties of $FeSe_{0.5}Te_{0.5}$ single crystals grown under different conditions. *The European Physical Journal B* **79**, 289-299, doi:10.1140/epjb/e2010-10473-5 (2011).
7. Zhang, P. *et al.* Observation of topological superconductivity on the surface of an iron-based superconductor. *Science* **360**, 182-186, doi:doi:10.1126/science.aan4596 (2018).
8. Tinkham, M. *Introduction to superconductivity*. (Courier Corporation, 2004).
9. Yuan, N. F. Q. & Fu, L. Zeeman-induced gapless superconductivity with a partial Fermi surface. *Physical Review B* **97**, 115139, doi:10.1103/PhysRevB.97.115139 (2018).
10. Agterberg, D. F., Brydon, P. M. R. & Timm, C. Bogoliubov Fermi Surfaces in Superconductors with Broken Time-Reversal Symmetry. *Physical Review Letters* **118**, 127001, doi:10.1103/PhysRevLett.118.127001 (2017).
11. Phan, D. *et al.* Detecting Induced $p \pm ip$ Pairing at the Al-InAs Interface with a Quantum Microwave Circuit. *Physical Review Letters* **128**, 107701, doi:10.1103/PhysRevLett.128.107701 (2022).
12. Zhu, Z. *et al.* Discovery of segmented Fermi surface induced by Cooper pair momentum. *Science* **374**, 1381-1385, doi:doi:10.1126/science.abf1077 (2021).
13. Hu, L.-H., Liu, C.-X. & Zhang, F.-C. Topological Larkin-Ovchinnikov phase and Majorana zero mode chain in bilayer superconducting topological insulator films. *Communications Physics* **2**, 25, doi:10.1038/s42005-019-0126-8 (2019).
14. Dimitrova, O. g. & Feigel'man, M. V. Theory of a two-dimensional superconductor with broken inversion symmetry. *Physical Review B* **76**, 014522, doi:10.1103/PhysRevB.76.014522 (2007).
